# EXPERIENCES OF DISCRIMINATION ARE ASSOCIATED WITH DECREASED FUNCTIONAL ABILITY IN AFRICAN IMMIGRANT OLDER ADULTS

**DOI:** 10.1093/geroni/igz038.2622

**Published:** 2019-11-08

**Authors:** Manka Nkimbeng, Karen Bandeen-Roche, Hae Ra Han, Sarah L Szanton, Roland Thorpe

**Affiliations:** 1 Johns Hopkins School of Nursing, Baltimore, Maryland, United States; 2 Johns Hopkins Bloomberg School of Public Health, Baltimore, Maryland, United States

## Abstract

Discrimination impacts functional health outcomes of African Americans and other racial/ethnic minorities in the United States; yet this is understudied in African immigrants whose population has risen by 137% since 2000. We examined the relationship between discrimination and physical function with a convenience sample of first-generation African immigrants age 50+ recruited through community-based organizations (N=124). Discrimination was measured with the Everyday Discrimination scale with higher scores indicating more experiences of discrimination (range=0-23). High versus low levels of discrimination were categorized at the mean. Physical function was measured using the PROMIS Physical Function measure with high scores indicating greater functional ability (range=11-50). Raw function scores were converted to standardized T-scores with a population mean of 50 and standard deviation (SD) of 10. Linear regression was used for analyses. Mean age of the sample was 61.4(SD=7.9) years. About two-thirds (63%) were female, more than half (52.4%) immigrated in search of better opportunities and half of the sample had high levels of discrimination. The mean function score was 44.2(SD=8.3) indicating that this sample had functional ability 6 points less than the population average. After adjusting for demographic and migration factors, the mean physical function score was 2.5 points lower (b=-2.53, 95% CI= -5.04, -0.01) for participants with more experiences compared to those with fewer experiences of discrimination. In conclusion, discrimination was associated with poor physical function in African immigrant older adults after adjusting for covariates. Longitudinal studies of discrimination and physical functioning should be pursued in more diverse, larger samples of African immigrants.

